# Longitudinal association between interparental conflict and risk-taking behavior among Chinese adolescents: testing a moderated mediation model

**DOI:** 10.1186/s13034-023-00556-4

**Published:** 2023-01-10

**Authors:** Wan-Yu Ye, Kai Dou, Lin-Xin Wang, Xiao-Qi Lin, Ming-Chen Zhang

**Affiliations:** 1 Department of Psychology and Research Center of Adolescent Psychology and Behavior, School of Education, Guangzhou University, Guangzhou Higher Education Mega Center, 230, Waihuan Road West, Panyu District, Guangzhou, People’s Republic of China; 2 Beijing Key Laboratory of Applied Experimental Psychology, National Demonstration Center for Experimental Psychology Education (Beijing Normal University), Institute of Developmental Psychology, Beijing Normal University, Beijing, People’s Republic of China; 3 Jieyang NO.1 High School Rongjiang New Town Campus, Student development center, Rongjing Road, Yuhu Town, Rongcheng District, Jieyang, Guangdong Province, People’s Republic of China

**Keywords:** Adolescence, Interparental conflict, Risk-taking behavior, Deviant peer affiliation, School climate

## Abstract

**Background:**

The interparental conflict has been associated with an increased adolescents’ engagement in risk-taking behaviors. However, few studies have examined the potential mediation of deviant peer affiliation and the potential moderation of school climate. Grounded in the ecological system theory, this study aimed to explore the mediating role of deviant peer affiliation and the moderating role of school climate between the association of interparental conflict and risk-taking behavior.

**Methods:**

This study conducted a longitudinal design (3 time points, 3 months apart) with the sample comprising 550 middle school students in southeastern China (52.91% males; mean age at Time 1 = 15.37). The performed measurements encompassed interparental conflict (T1), deviant peer affiliation (T2), school climate (T3), risk-taking behavior (T1/T2/T3), and demographic information.

**Results:**

The moderated mediation model revealed that after controlling for T1/T2 risk-taking behavior, T1 interparental conflict was longitudinally and positively correlated with T3 risk-taking behavior through T2 deviant peer affiliation. Furthermore, moderated mediation analysis demonstrated that a positive school climate ameliorated the adverse impact of deviant peer affiliation on risk-taking behavior, thereby mitigating the indirect effect of interparental conflict on risk-taking behavior among adolescents.

**Conclusions:**

Our findings propose a nuanced explanation of the processing mechanisms between interparental conflict and risk-taking behaviors among Chinese adolescents. The theoretical and practical implications of the findings are discussed.

**Supplementary Information:**

The online version contains supplementary material available at 10.1186/s13034-023-00556-4.

## Background

Adolescence is the time when the majority of people are more likely to engage in risk-taking behaviors, which are socially unacceptable behaviors with potential costs and may create psychological, social, and school difficulties [3, 30]. The phenomenon of risk-taking behavior (e.g., violent crime, alcohol abuse, risky driving, unsafe sex) among adolescents has attracted much public concern and the attention of researchers [10, 48]. Substantial evidence suggests that risk-taking behavior is detrimental to adolescents’ physical and mental health [31, 38]. For effective prevention and remediation programs, it is imperative to identify risk factors and underlying mechanisms for risk-taking behavior among adolescents.

Based on Bronfenbrenner's [14] ecological model, the family is the most influential and immediate aspect of the ecological environment in terms of human development. A negative family environment may result in maladaptive developmental outcomes [64, 82]. Inspired by this theory, numerous studies have examined the role of family factors in adolescent risk-taking behavior [28, 32, 60, 84]. Remarkably, interparental conflict (IPC) plays a particularly essential role among these factors. IPC refers to physical aggression or verbal conflict between parents due to disagreement or other reasons [57]. Stress-coping theory proposes that adolescents who experience IPC seek risk-taking behavior (e.g., substances) to cope with their stress [68, 77]. Additionally, parents who are bothered by their conflicts may be too distracted by these issues to supervise and monitor their adolescents, resulting in the adolescent having more opportunities to engage in risk-taking behavior [60]. Consistent with the above-mentioned views, several studies have shown that IPC positively predicted risk-taking behavior [22, 35, 45, 83]. For instance, a meta-analytic study found that IPC was a significant factor in risk-taking behavior [83]. Similarly, in a longitudinal study, Davies et al. [22] suggested that adolescents’ perceptions of IPC significantly predicted their externalizing difficulties three years later, including risk-taking behavior (cheating, stealing, fighting with others).

Evidence of the direct link between IPC and risk-taking behavior has been demonstrated in previous studies [21, 24], but the underlying mechanisms that could *account for* this link (i.e., mediating mechanisms) and *alter* it (i.e., moderating mechanism) remain largely unexplored. Peer and school contexts are critical settings for adolescents’ behavior development according to the ecological model [14]. Adolescents who are exposed to IPC may apply the conflictual communication strategies (e.g., assault, abuse) learned in their family to peer interactions and affiliate with deviant friends, which in turn is related to problem behaviors [23]. In addition, a positive school context (i.e., school climate) may buffer the impacts of risk factors in another context on adolescents’ development outcomes [46]. Hence, the present study employed a complex moderated mediation model to offer a more nuanced understanding of real-world phenomena. The first goal was to examine deviant peer affiliation as a potential and underexplored mediator in the link between IPC and risk-taking behavior. The second goal was to investigate whether this mediating process is moderated by school climate.

## The mediation effect of deviant peer affiliation

Individuals are especially vulnerable to peer influence during adolescence, and the choices of friends affect their behavioral development [54]. Deviant peer affiliation refers to adolescents' selective association with peers who violate rules, social ethics, and laws [72]. Numerous studies have shown that deviant peer affiliation is a crucial predictor of adolescent deviant behavior [62, 94]. Social learning theory suggests that adolescents learn more risk-taking behavior through observation and imitation when socializing with deviant peers [5, 81, 86]. Specifically, adolescents may be pressured by their peers to conform to deviant peer norms [55]. Furthermore, adolescents obtain support and defend their high position in the deviant peer’s status hierarchy by showing risk-taking behavior, such as alcoholism, and smoking [20]. In line with these notions, plentiful evidence has suggested that deviant peer affiliation increases risk-taking behavior [33, 52, 95, 96].

Family and peers are two such micro-systems found to directly, indirectly, and interactively affect multiple aspects of adolescent development according to the ecological system theory [14]. It is plausible to speculate that IPC may increase deviant peer affiliation among adolescents. Previous studies indicated that destructive IPC threatens adolescents' sense of safety and support from their families, which may further create conditions conducive to adolescents seeking to affiliate with deviant peers [11, 59]. Additionally, adolescents with dysfunctional relationships between parents are more likely to experience worries and fears about the peer context, leading to decreased peer support and increased loneliness and engagement with deviant peers [90]. Similarly, several longitudinal studies have also demonstrated that IPC is a risk factor for adolescents associated with deviant peers [60, 63]. Taken together, IPC may be indirectly associated with risk-taking behavior through deviant peer affiliation.

## The moderation effect of school climate

School is a central environment that the vast majority of adolescents interact with daily [14, 85], which may operate together with deviant peer affiliation to explain why IPC is associated with risk-taking behavior. Specifically, school climate is a lasting and stable environmental characteristic and can reflect all aspects of the school experience of adolescents [89]. A longitudinal study demonstrated that the normative standards of behavior that adolescents perceive decrease and risk-taking behavior tends to increase under a negative school climate [27]. In addition, adolescents’ negative perceptions of school climate were associated with increased psychological and behavioral difficulties [78]. More importantly, according to the ecological systems theory [14], negative connections between microsystems may occur with negative consequences. This is, a negative school climate may operate as a risk factor and strengthen the deleterious effects of deviant peer affiliation on adolescents’ risk-taking behavior [6, 56].

On the contrary, positive factors in one environment may buffer the impacts of risk factors in another environment on adolescents’ development outcomes, according to the stress-buffering model [18]. Consistent with this model, we consider that a positive school climate would moderate the association between IPC and risk-taking behavior. Previous studies have proved that a positive school climate meets the psychological needs of adolescents and reduces risk-taking behavior [79, 97]. Moreover, adolescents can obtain emotional support and help from teachers in a positive school climate,with this, adolescents may reduce the possibility of adopting negative stress coping strategies (e.g., risk-taking behavior), and better navigate the stress brought by deviant peer affiliation [58, 66].

Thus, different school climates might be an important variable for differences in adolescents’ risk-taking behavior. Based on the previous research, we propose that a negative school climate may facilitate the intensified effect of deviant peer affiliation on risk-taking behavior, while a positive school climate may attenuate this relation.

## The current study

**Fig. 1:**
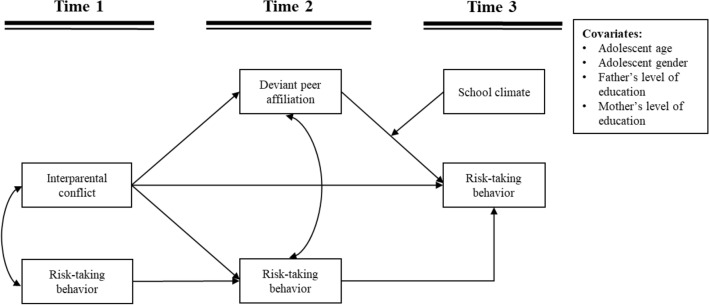
Conceptual moderated mediation model

Environment factors, such as family, peer, and school context play a role in adolescent risk-taking behavior. Based on the perspective of ecological system theory, this study comprehensively considers family, peers, and school’s environmental perspectives to reveal the mechanism that affects adolescents' risk-taking behavior. Specifically, the present study explored the longitudinal relations between IPC and risk-taking behavior in a Chinese sample, and the mechanism of deviant peer affiliation and school climate. Using three-wave of longitudinal data, separated by three months, we tested a longitudinal model that includes the processes depicted in Fig. 1. Interparental conflict (T1), deviant peer affiliation (T2), school climate (T3), risk-taking behavior (T1/T2/T3) were collected using a self-reported questionnaire. Additionally, we also included adolescent age, adolescent gender, father’s level of education and mother’s level of education as control variables in our analyses.

We hypothesized that: IPC is associated positively with adolescent risk-taking behavior (Hypothesis 1); deviant peer affiliation will mediate the association between IPC and risk-taking behavior (Hypothesis 2); school climate would moderate the association between deviant peer affiliation and risk-taking behavior (Hypothesis 3a), and school climate would moderate the mediation effect of deviant peer affiliation (Hypothesis 3b).

## Method

### Participants and procedure

We used a convenience sampling method to recruit 550 adolescents (291 males and 259 females) from one junior high school (*N* = 209, age-range: 12 ~ 15 years, *M*_age_ = 13.81 years old, *SD* = 0.68, 89 females) and two senior high schools (*N* = 341, age-range: 15 ~ 18 years, *M*_age_ = 16.33 years old, *SD* = 0.49, 170 females) in Guangzhou, China. In terms of grade distribution, 100 students were from grade 7, 109 from grade 8, 204 from grade 10, and 137 from grade 11. In Time 1 (T1; September 2018), the adolescents were between 12 and 18 years old (*M*_age_ = 15.37, *SD* = 1.35). The majority of participants’ parents had high school degree, 39.8% of the fathers had a middle school degree, 47.4% had earned a college degree or equivalent, and 9.0% had earned a graduate degree; 44.4% of the mothers had a middle school degree, 44.2% had earned a college degree or equivalent, and 5.4% had earned a graduate degree. Three months later (T2, December 2018) we used the same procedure and 97.3% of the initial sample (*N* = 535, 279 males and 256 females). Another three months later (T3, March 2019), 513 adolescents (269 males and 244 females) remained in the study (attrition rate = 6.7%). The attrition at each time point was because adolescents were absent on the day of data collection. Additionally, all participants attended their original schools during the longitudinal study period.

Ethical approval was obtained from the ethics committee of the corresponding author’s affiliation (Protocol Number: GZHU 2019017). We received oral and written consent from all participants and parents’ signed consent before data collection. Before signing informed consent, all participants were aware of the right to freely drop out of the study at any time with no further obligations. Procedures following a standardized protocol, trained research assistants collected data in the classroom during regular school hours. This procedure was repeated for all collection time points. After completing the study, no rewards were offered for their participation.

### Measures

#### Interparental conflict

The IPC was assessed by the Interparental Conflict subscale from the Children’s Perception of Interparental Conflict questionnaire at T1 [16, 49]. The IPC questionnaire consists of 7 items that evaluate the frequency of IPC. Participants responded on a four-point scale ranging (from 1 = *never* to 4 = *very often*). The IPC scores were the total scores divided by the number of items, with higher scores indicating more frequent IPC. Sample items included “How often do your parents threaten each other when they disagree”. The Chinese version of the IPC presented good internal consistency [91]. Cronbach’s alpha value for the scale was 0.82 in the current study.

#### Deviant peer affiliation

Deviant peer affiliation was measured at T2 using the ten-item Deviant Peer Affiliation Questionnaire [7, 17]. Participants reported the number of friends who engaged in certain deviant behavior (e.g., cheating in exams, gambling, or stealing) on a 5-point scale ranging (from 1 = *none* to 5 = *more than six*). For each participant, scores for each item were summed and divided by the total number of items to arrive at a mean deviant peer affiliation score, a higher mean score indicated that participants affiliate with more deviant peers. The Chinese version of the deviant peer affiliation has demonstrated good internal consistency in adolescents [28]. The Cronbach’s alpha in this study was 0.86.

#### School climate

Participants’ perceived school climate was assessed by the school climate questionnaire at T3 [8, 9]. This scale consists of 28 items that evaluate seven dimensions of school climate: teacher-student relations (5 items), student–student relations (4 items), student engagement-school-wide (5 items), clarity of expectations (4 items), fairness of rules (4 items), school safety (3 items), and bullying school-wide (3 items). Sample items are “School rules are fair” and “Teachers care about their students”. These items were rated on a four-point scale (from 1 = *very inconsistent* to 5 = *very consistent*). The school climate scores were calculated as the sum of the item scores divided by the total number of items, a higher score indicated that participants perceive a positive school climate. The Chinese version of the school climate has demonstrated good internal consistency in adolescents [87]. The Cronbach’s alpha of this scale was 0.94 in the present study.

#### Risk-taking behavior

Participants completed the Adolescent Risk-taking Questionnaire (ARQ) at T1, T2, and T3 [41], an 11-item questionnaire that was used to measure how often participants had engaged in risk-taking behavior (e.g., drugging, unsafe sex, alcoholism), using a five-point scale ranging from 0 = *never,* to 4 = *always*. The scale consists of three dimensions: rebellious behaviors (6 items; e.g., “Smoking”), reckless behaviors (2 items; e.g., “Having unprotected sex”), and antisocial behavior (4 items; e.g., “Teasing and picking on people”). The ARQ scores were the total scores divided by the number of items, and higher scores indicated more frequent risk-taking behavior. The Chinese version of ARQ showed good internal consistency [48]. Cronbach’s alpha values for the scale were 0.80, 0.84, and 0.83 at Time 1, Time 2, and Time3, respectively.

#### Demographic covariates

Adolescent age, adolescent gender (1 = *males*; 2 = *females*), as well as father’s and mother’s education (1 = *primary school and below*; 2 = *middle school degree*; 3 = *undergraduate degree or equivalent*; 4 = *graduate degree*) were included as demographic covariates in all the analyses because their significant associations with risk-taking behavior have been documented in previous studies [29, 34].

### Analytic strategies

A total of 6.7% of the data were missing across three-time points, and missing data were addressed using full information maximum likelihood (FIML) under the missing-at-random assumption in our main analysis [1]. First, attrition analysis, descriptive statistics and bivariate correlations analysis were conducted for all the variables of interest with IBM SPSS 26.0. Second, we examined whether common method bias would be a salient concern, given the use of self-report measures. Third, we performed a structural equation modeling (SEM) by M*plus* 8.3 [65]. To test hypotheses 1 and 2, IPC at T1 and adolescent risk-taking behavior at T3 were included in the mediation model as the independent and dependent variables, respectively, whereas deviant peer affiliation at T2 served as the mediator. Then, we integrated the moderator (i.e., T3 school climate) into the aforementioned mediation model to test hypotheses 3a and 3b. For the moderated-mediation model, all variables were centered before generating the interaction terms. When the interaction effect was significant, we further conducted the simple slope analysis and tested the mediating effect of T2 deviant peer affiliation when levels of the moderator (i.e., T3 school climate) were one standard deviation below and above the mean. This allowed us to examine the extent to which school climate would moderate the association between T2 deviant peer affiliation and T3 risk-taking behavior and to examine the extent to which school climate would moderate the mediation effects of deviant peer affiliation.

Model adequacy was evaluated with the following indices: the values of comparative fit index (CFI; acceptable > 0.90), root mean square error of approximation (RMSEA; acceptable < 0.08; [75], and standardized root mean square residual (SRMR,acceptable < 0.08). Considering that bootstrapping has several advantages over traditional approaches in examining mediation models [70], we used bootstrapping technique (*N* = 5000) and its 95% confidence intervals (CI) to assess the indirect and direct effects. When zero was not included in the 95% CI, the (moderated) mediation effect would be deemed to be tenable.

## Results

### Attrition analysis

Attrition analyses was used to examine potential bias between participants who had completed measures across time points (Group1) and participants who dropped out at T2 and/or T3 (Group2). The results showed that the two groups did not differ in age (*t* (548) = 0.98, *p* = 0.326), T1 interparental conflict (*t* (548) = 0.72, *p* = 0.469), T2 deviant peer affiliation (*t* (533) = -1.93, *p* = 0.054), T1/T2/T3 risk-taking behavior (*t* (541) = 0.16, *p* = 0.873; *t* (531) = -0.73, *p* = 0.467; (*t* (517) = 0.28, *p* = 0.778), T3 school climate (*t* (526) = 0.49, *p* = 0.625), gender (*χ*^*2*^(1) = 0.683, *p* = 0.409). However, the two groups differed in mother’s education (*χ*^*2*^(3) = 16.153, *p* = 0.001) and father’s education (*χ*^*2*^(3) = 12.328, p = 0.006). With the attrition group containing lower proportion of parents with “primary school and below” and higher proportion of “undergraduate” education. Based on Little’s Missing Completely at Random (MCAR) test we could not conclude that the data were MCAR (*χ*^2^(38) = 70.703, *p* = 0.001). Thus, we created a variable indicating whether or not a participant had missing data, and correlated this variable with main outcome variables. The correlations between missingness and these outcome variables were small and non-significant. The results suggested that the data can be treated as missing at random (MAR). In summary, these results indicated that our data set was unlikely to be biased due to attrition.

### Common method bias

Because all of the questions were filled out by adolescents, common method bias was a potential issue [69]. To determine if common method bias might pose a threat to the interpretation of our findings, we used both exploratory and confirmatory procedures. First, the single-factor analysis approach of Harman was used to check common method bias [42]. The result indicated that the first factor only explained 17.97% of the variance, which is less than the 50% threshold. This affirms the absence of the common method bias. Second, confirmatory factor analysis (CFA) was used to investigate the factor confirmation. We conducted CFA to contrast the six-factor model (based on the six main study variables) and the one-factor model (including all self-assessment items). The resulting six-factor model produced acceptable fit indices: Normed Chi-square (*χ*^2^/df) = 4.094 is less than 5 [44]. Tucker-Lewis Index (TLI) = 0.940, and Comparative Fit Index (CFI) = 0.942 are above 0.90 [4]. The Root Mean Square Error of Approximation (RMSEA) value is 0.075, which is lower than 0.08 [15]. Additionally, one-factor model had poor model fit: *χ*^2^/df = 28.702, TLI = 0.463, CFI = 0.476, RMSEA = 0.224. As a result, common method bias did not present to pose a threat in interpreting our results.

### Descriptive and correlations

Descriptive statistics and correlations among primary study variables are presented in Table 1. Specifically, T1 IPC was negatively related to T3 school climate and positively related to T2 deviant peer affiliation, and T1/T2/T3 risk-taking behavior, respectively. Besides, T2 deviant peer affiliation was negatively related to T3 school climate and positively related to T1/T2/T3 risk-taking behavior, respectively. Moreover, T3 school climate was negatively related to T1/T2/T3 risk-taking behavior, respectively.

**Table 1:** The means, standard deviations, correlations among the variables

	*M*	*SD*	1	2	3	4	5	6	7	8	9
Covariates											
1. Student age at T1	15.37	1.35									
2. Student gender	47.56^a^	–	.05								
3. Father’s level of education	–	–	− .05	.05							
4. Mother’s level of education	–	–	− .08	.06	.61^***^						
Key variables											
5. T1 Interparental conflict	1.10	0.47	− .10^*^	.05	− .02	− .06					
6. T2 Deviant peer affiliation	1.60	0.66	− .03	− .02	.05	.05	.11^*^				
7. T3 School climate	3.07	0.43	.09^*^	.01	− .01	− .01	− .19^***^	− .29^***^			
8. T1 Risk-taking behavior	0.39	0.34	− .03	− .09^*^	.05	.01	.20^***^	.35^***^	− .15^**^		
9. T2 Risk-taking behavior	0.42	0.38	− .06	− .04	.07	.02	.16^***^	.40^***^	− .22^***^	.49^***^	
10. T3 Risk-taking behavior	0.41	0.36	− .05	− .04	.03	.01	.24^***^	.39^***^	− .26^***^	.48^***^	.63^***^

Sample size ranged from 513 to 550 due to missing data. ^*^ *p* < .05, ^**^ *p* < .01, ^***^p < .001. Student gender: 1 = males, 2 = females; Education: 1 = primary school, 2 = middle school, 3 = undergraduate, 4 = graduate student; ^a^ The percentage of female adolescents; T1 = Time 1, T2 = Time 2, T3 = Time 3

### Mediating effects of deviant peer affiliation

**Fig. 2:**
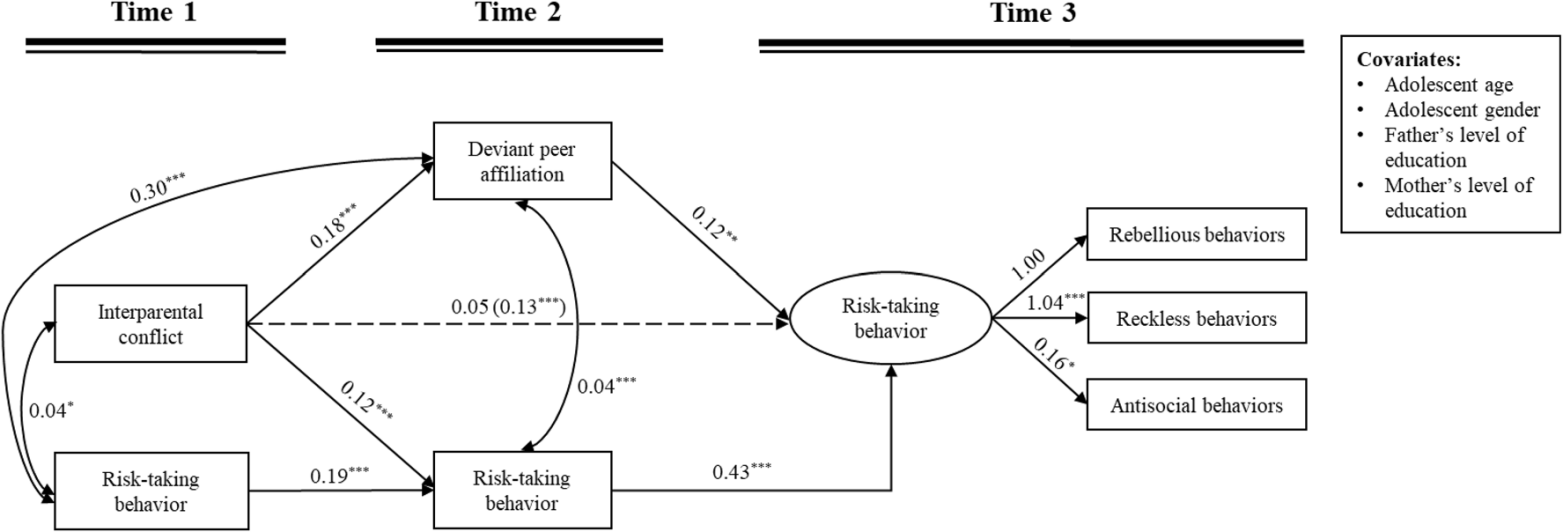
The mediating effect of T2 deviant peer affiliation in the relation between T1 interparental conflict and T3 risk-taking behavior. Unstandardized coefficients are reported; ^*^p < 0.05, ^**^p < 0.01, ^***^p < 0.001; T1 = Time 1, T2 = Time 2, T3 = Time 3; The value in parenthesis represent the direct effect, before incorporating mediation into the model. Dashed line indicates a non-significant coefficient

The mediation model (Table 2, Fig. 2) revealed a good fit to the data: χ^2^ = 90.95, *df* = 23, *p* < 0.001; RMSEA = 0.073 with a 90% CI, [0.058, 0.089]; CFI = 0.943 and SRMR = 0.041. After controlling for covariates and T1/T2 risk-taking behavior, T1 IPC was significantly related with T2 deviant peer affiliation (*B* = 0.18, *SE* = 0.05, *p* < 0.001), but insignificantly related with T3 risk-taking behavior (*B* = 0.05, *SE* = 0.03, *p* = 0.063). Results of the mediation analyses indicated that the mediation effect of T2 deviant peer affiliation was significant (*B* = 0.02, *SE* = 0.01, 95%CI = [0.008, 0.046]).

**Table 2:** Summary of the direct and indirect effects

Direct and indirect effects	Bias-corrected bootstrapped estimates for the effects			
	*b*	*SE*	95% CI	β
Direct pathway				
T1 Interparental conflict → T3 Risk-taking behavior	**.05**	**.03**	**[.003, .108]**	**.09**
Indirect pathways				
T1 Interparental conflict → T2 Deviant peer affiliation → T3 Risk-taking behavior	**.02**	**.01**	**[.008, .046]**	**.04**

T1 = Time 1, T2 = Time 2, T3 = Time 3; The significant results are in bold. According to Preacher and Kelley [71], standardized indirect effects around 0.01 were “small”, effects around 0.09 were “medium”, and effect around 0.25 were “large”. *b = *unstandardized coefficient, *SE * = standard error, *CI* = confidence interval for the standardized coefficient, *β* = standardized coefficient

### Moderating effects of school climate

**Fig. 3:**
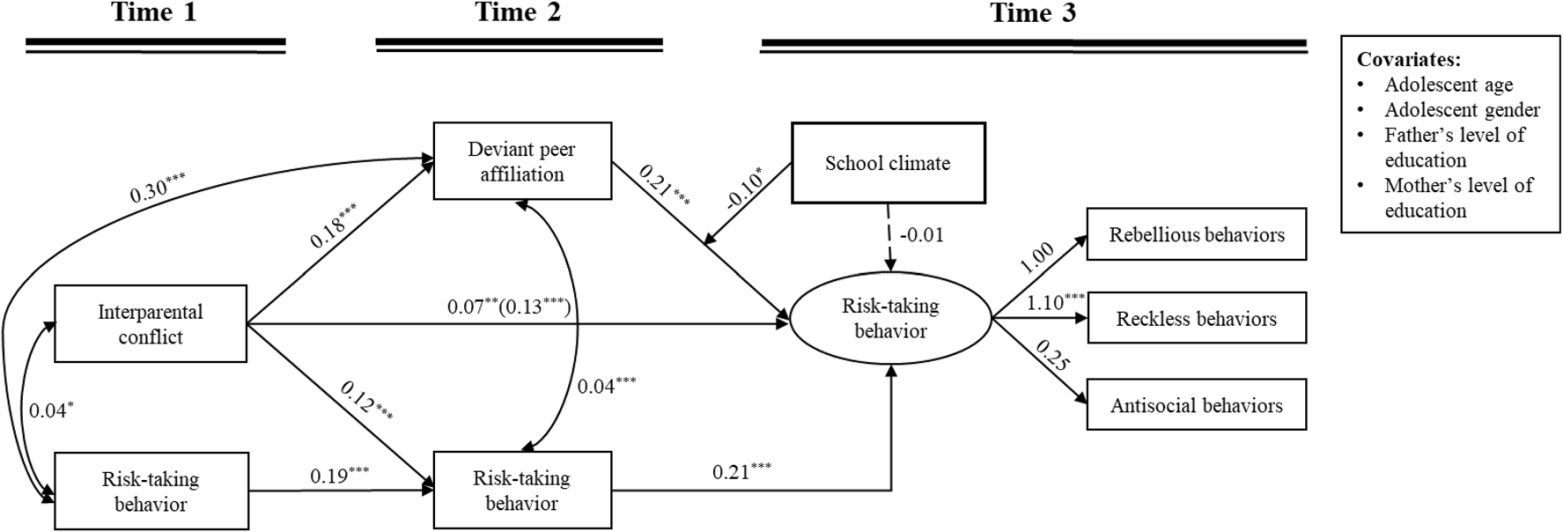
Moderated mediation model. Unstandardized coefficients are reported; ^*^p < 0.05, ^**^p < 0.01, ^***^p < 0.001; T1 = Time 1, T2 = Time 2, T3 = Time 3; The value in parenthesis represent the direct effect, before incorporating mediation into the model. Dashed line indicates a non-significant coefficient

Based on the testing for the mediation model, we continued to examine whether T3 school climate would moderate the relation between T2 deviant peer affiliation and T3 risk-taking behavior as well as the mediating effect of T2 deviant peer affiliation. The moderated mediation model implied a good fit to the data: *χ*^2^ = 129.31, *df* = 29, *p* < 0.001; RMSEA = 0.079 with a 90% CI = [0.066, 0.093]; CFI = 0.919 and SRMR = 0.034. The results are displayed in Table 3, which suggested that T3 school climate moderated the relation between T2 deviant peer affiliation and T3 risk-taking behavior (*B* = -0.10, *SE* = 0.04, *p* = 0.017, β = -0.10). Subsequently, the follow-up simple slope analysis (Fig. 3) revealed that the relation T2 deviant peer affiliation and T3 risk-taking behavior was stronger at the negative T3 school climate (*B* = 0.05, *SE* = 0.02, *p* < 0.05) than the positive T3 school climate (*B* = 0.02, *SE* = 0.02, *p* = 0.210).

**Table 3:** Summary of the moderated mediation model

	T2 Deviant peer affiliation (*R*^2^ = 0.03)					T2 Risk-taking behavior (*R*^2^ = 0.19)					T3 Risk-taking behavior (*R*^2^ = 0.48)			
	*B*	*SE*	β	*p*		*B*	*SE*	β	*p*		*B*	*SE*	β	*p*
Covariates														
Student age at T1											**.02**	**.01**	**.06**	**.019**
Student gender											− **.06**	**.03**	− **.07**	**.017**
Father’s level of education											.00	.02	− .01	.888
Mother’s level of education											− .01	.03	− .01	.720
Key variables														
T1 Interparental conflict	**.18**	**.05**	**.09**	** < .001**		**.12**	**.02**	**.21**	** < .001**		**.07**	**.03**	**.09**	**.004**
T2 Deviant peer affiliation											**.21**	**.02**	**.31**	** < .001**
T3 School climate											− .01	.02	− .02	.541
T2 Deviant peer affiliation × T3 School climate											− **.10**	**.04**	− **.10**	**.017**
T1 Risk-taking behavior						**.19**	**.03**	**.34**	** < .001**					
T2 Risk-taking behavior											**.21**	**.02**	**.16**	** < .001**

Student gender: 1 = males, 2 = females; Education: 1 = primary school, 2 = middle school, 3 = undergraduate, 4 = graduate student; T1 = Time 1, T2 = Time 2, T3 = Time 3; The significant results are in bold

**Fig. 4:**
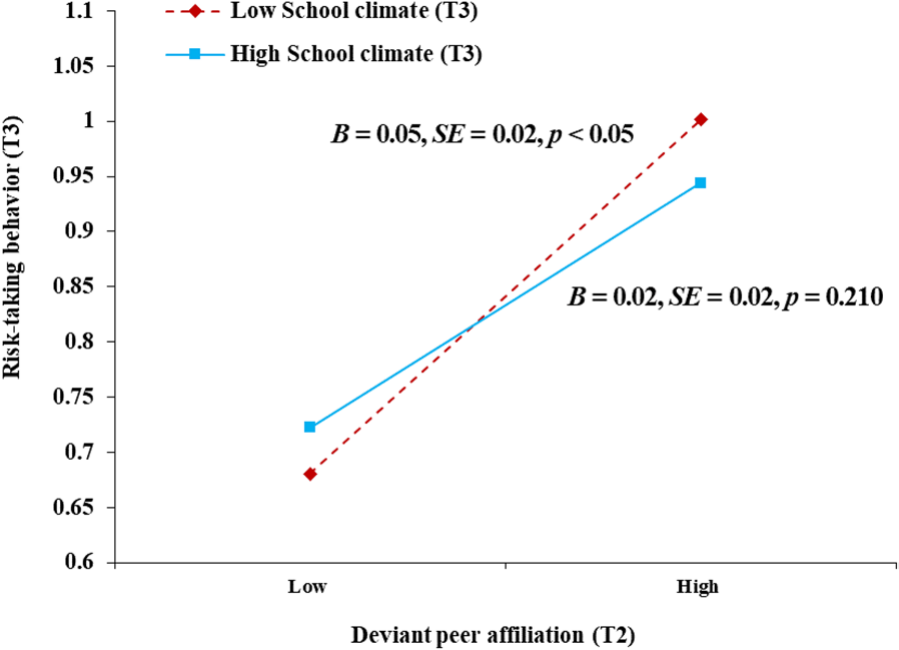
The relation between T2 deviant peer affiliation and T3 risk-taking behavior by T3 school climate. T2 = Time 2, T3 = Time 3

As illustrated in Fig. 4 and Table 4, the moderated mediation model indicated that the indirect effect of T2 deviant peer affiliation was significantly stronger when T3 school climate was lower (*B* = 0.05, *SE* = 0.01, 95% CI = [0.021, 0.072]) than when T3 school climate was high (*B* = 0.03, *SE* = 0.01, 95% CI = [0.015, 0.055]). In summary, we came to conclude that IPC has a negative stronger association with adolescents’ risk-taking behavior via deviant peer affiliation in a negative school climate.

**Table 4:** Conditional indirect effects of T1 interparental conflict on T3 risk-taking behavior via T2 deviant peer affiliation by levels of T3 school climate

Levels of T3 school climate	*B*	*SE*	95% CI
Low (*M − SD*)	**.05**	**.01**	**[.021, .072]**
Med (*M*)	**.04**	**.01**	**[.019, .062]**
High (*M* + *SD*)	**.03**	**.01**	**[.015, .055]**
Diff (High − Low)	− **.01**	**.01**	**[**− **.032, **− **.004]**

T1 = Time 1, T2 = Time 2, T3 = Time 3. The significant results are in bold

## Discussion

Based on the ecological system theory perspective [14], the present study examined the longitudinal association between IPC and risk-taking behavior among Chinese adolescents and also investigated the underlying mediating and moderating mechanisms. Moreover, the multiple development contexts (i.e., family and school) better reveal the developmental courses of adolescents’ risk-taking behavior compared with a single development context. The primary findings of this study were that deviant peer affiliation mediated the association between IPC and risk-taking behavior, especially in a negative school climate. Additionally, this study used a short-term longitudinal design to measure the core variables. This procedure addressed previous research focused primarily on cross-sectional methodology, and thus is conducive to causal inference and control for common method bias. Such a design has been widely used in previous research [12, 19, 87].

Firstly, we proved hypothesis 1 that IPC was positively associated with adolescent risk-taking behavior after controlling for the baseline levels of risk-taking behavior. This finding was consistent with previous studies [2, 36, 37, 50]. IPC might signify inadequate family support resources and poor parent–child relationships [61]. This may result in adolescents’ risk-taking behavior (e.g., substance use) to help them relieve stresses from IPC [22, 32, 84]. Moreover, social learning theory noted that adolescents observe and learn from particular expressions or behavioral models of IPC, which in turn increase the risk of behavioral problems [5, 47]. As can be seen, parents remain to play a vital role in adolescents’ development.

Secondly, we found that deviant peer affiliation mediated the relation between the IPC and adolescent risk-taking behavior. In other words, IPC increases adolescent risk-taking behavior by contributing to deviant peer affiliation. Our findings are consistent with our hypothesis 2 and previous studies [13, 26, 67]. Particularly, the first link of the mediational chain supports several studies [43, 73] documenting that IPC was positively associated with deviant peer affiliation. Davies et al. [23] suggested that adolescents who are unable to get security and warmth from their families are more inclined to seek support and belongingness from peers and are susceptible to social difficulties such as deviant peer affiliation. Following the social learning theory, adolescents may learn negative interpersonal and communication skills from IPC, which may affect adolescents’ choices of friends and peer groups and prompt them to choose an adverse peer context [5, 63]. The second link of the mediation model is also consistent with previous research [20, 93, 95]. Adolescence is a crucial period when individuals are particularly susceptible to peer influence [76]. To gain peer acceptance and support, adolescents may engage in more peer-led risk-taking behavior [39]. Moreover, peer pressure and reinforcement may also raise the likelihood of developing risk-taking behavior [74].

Another important contribution of our findings was that the mediation effect of deviant peer affiliation was moderated by school climate. In comparison to the positive school climate, the negative school climate enhanced the adverse effect of deviant peer affiliation on risk-taking behavior. Our findings are in line with the previous research which demonstrated that negative school context facilitated the deteriorative effect of deviant peer affiliation on adolescent development [46, 89, 92]. One potential explanation is that the negative school climate can meet the needs of affiliating with deviant peers, and send a message to the student that their behavior may not be supervised by school rules, thereby reinforcing risk-taking behavior [78]. In addition, our findings do not support the stress-buffering model, the protective effect of the positive school climate seems to attenuate as the level of deviant peer affiliation increases. Concerning this, DeLay et al. [25] demonstrated that friendship choices have a lasting impact on adolescent deviance, adolescents who choose to affiliate with deviant peers are less able to capitalize on the benefit of the positive context and may engage in more risk-taking behavior. Taken together, the present study contributes to the existing research and highlights the importance of the microsystem (i.e., negative school climate) in enhancing the negative effect between deviant peer affiliation and risk-taking behavior. In creating prevention and intervention strategies, the importance of school climate should be considered.

Although this study provides important information about the underlying factors associated with risk-taking behavior in Chinese adolescents, several limitations still need to be mentioned. First, the sample in this study was predominantly Chinese adolescents recruited from a large metropolitan area and did not include rural and small cities group and special groups (e.g., left-behind children). Future work is required for the representativeness of the sample. Second, although this study used a three-point longitudinal design, the findings are still correlational and do not suggest causality. Moreover, we measured IPC, deviant peer affiliation, and school climate only at a one-time point, baseline of deviant peer affiliation and school climate were not controlled. Future research should carefully consider the timing of effects and the potential benefits of cross-lagged designs. Finally, the only adolescent report was used to collect data. Although adolescents are the best reporters of their perceptions of IPC and school climate [40, 51], future research should use multiple informants (e.g., parent report, peer report, teacher report) to provide a more rigorous test for research hypotheses.

Despite these limitations, multiple theoretical and practical can be drawn to reduce adolescents’ risk-taking behavior. First, the impact of IPC on adolescents’ behavioral development is worthy of attention. Parents should avoid or reduce conflict to construct a harmonious family environment for adolescents. More importantly, given that IPC may increase the risk-taking behavior via deviant peer affiliation among adolescents, encouraging them to participate in positive peer interactions and reduce deviant peer affiliation should be noteworthy. Second, it’s critical to pay more attention to those adolescents who perceived a negative school climate from school, as well as to enhance teacher-student communication and support, which may help to reduce deviant peer influence and risk-taking behavior among adolescents [53, 80, 88]. Finally, our moderated mediation model demonstrated that adolescent risk-taking behavior is the joint effect of the risk factors from parents, peers, and school microsystems. Thus, it is necessary to comprehensively consider the multi-level risk and protection factors of family, peers, and school, rather than focusing on factors from only one aspect.

## Conclusions

This three-point longitudinal study examined the underlying mechanism of *how* and *for whom* IPC is related to risk-taking behavior in adolescents. Specifically, the present study demonstrates that IPC is associated with risk-taking behavior through deviant peer affiliation in Chinese adolescents. Furthermore, a positive school climate serves as a protective factor to alleviate the negative impact of deviant peer affiliation on adolescents.

## Supplementary Information

**Additional file 1:** Online supplementary files. Additional file 1 includes description and specific items for the four scales used in this study. Specifically, these include interparental conflict scale, deviant peer affiliation scale, school climate scale, and risk-taking behavior scale. Table 1. The means, standard deviations, correlation of school climate dimensions with the main variables. Table 2. Summary of the moderated mediation model (the model was constructed by using each of the 7 dimensions of school climate as moderating variables).

## Data Availability

The datasets used and/or analyzed during the current study are available from the corresponding author on reasonable request.

## References

[1] Acock AC (2005). Working with missing values. J Marriage Fam.

[2] Ai T, Xu Q, Li X, Li D (2017). Interparental conflict and Chinese adolescents’ suicide ideation and suicide attempts: the mediating role of peer victimization. J Child Fam Stud.

[3] Albert D, Steinberg L (2011). Judgment and decision making in adolescence. J Res Adolesc.

[4] Bagozzi RP, Yi Y (1988). On the evaluation of structural equation models. J Acad Mark Sci.

[5] Bandura A, Walters RH (1963). Social learning and personality development.

[6] Bao Z, Jiang Y, Zhu J, Zhang W (2020). School connectedness and deviant peer affiliation among Chinese adolescents: the mediating role of sleep problems. Curr Psychol.

[7] Bao Z, Li D, Zhang W, Wang Y (2015). School climate and delinquency among Chinese adolescents: analyses of effortful control as a moderator and deviant peer affiliation as a mediator. J Abnorm Child Psychol.

[8] Bear GG, Gaskins C, Blank J, Chen FF (2011). Delaware school climate survey-student: Its factor structure, concurrent validity, and reliability. J Sch Psychol.

[9] Bear GG, Yang C, Mantz L, Pasipanodya E, Hearn S, Boyer D. Technical manual for Delaware school survey: Scales of school climate, bullying victimization, student engagement, and positive, punitive, and social-emotional learning techniques. Delaware Positive Behavior Support (DE-PBS) and School Climate Transformation Projects; 2014

[10] Ben-Zur H, Zeidner M (2009). Threat to life and risk-taking behaviors: a review of empirical findings and explanatory models. Pers Soc Psychol Rev.

[11] Blakemore SJ (2018). Avoiding social risk in adolescence. Curr Dir Psychol Sci.

[12] Boer M, Stevens GWJM, Finkenauer C, de Looze ME, van den Eijnden RJJM (2021). Social media use intensity, social media use problems, and mental health among adolescents: investigating directionality and mediating processes. Comput Hum Behav.

[13] Bowman MA, Prelow HM, Weaver SR (2007). Parenting behaviors, association with deviant peers, and delinquency in African American adolescents: a mediated-moderation model. J Youth Adolesc.

[14] Bronfenbrenner U (1979). The ecology of human development.

[15] Browne MW, Cudeck R (1992). Alternative ways of assessing model fit. Sociol Methods Res.

[16] Buehler C, Krishnakumar A, Stone G, Anthony C, Pemberton S, Gerard J, Barber BK (1998). Interparental conflict styles and youth problem behaviors: a two-sample replication study. J Marriage Fam.

[17] Chen W, Li D, Bao Z, Yan Y, Zhou Z (2015). The impact of parent-child attachment on adolescent problematic internet use: a moderated mediation model. Acta Psychol Sin.

[18] Cohen S, Wills TA (1985). Stress, social support, and the buffering hypothesis. Psychol Bull.

[19] Cooper B, Eva N, Zarea Fazlelahi F, Newman A, Lee A, Obschonka M (2020). Addressing common method variance and endogeneity in vocational behavior research: a review of the literature and suggestions for future research. J Vocat Behav.

[20] Daspe ME, Arbel R, Ramos MC, Shapiro LAS, Margolin G (2019). Deviant peers and adolescent risky behaviors: the protective effect of nonverbal display of parental warmth. J Res Adolesc.

[21] Davies PT, Coe JL, Martin MJ, Sturge-Apple ML, Cummings EM (2015). The developmental costs and benefits of children's involvement in interparental conflict. Dev Psychol.

[22] Davies PT, Hentges RF, Coe JL, Martin MJ, Sturge-Apple ML, Cummings EM (2016). The multiple faces of interparental conflict: implications for cascades of children's insecurity and externalizing problems. J Abnorm Psychol.

[23] Davies PT, Martin MJ, Cummings EM (2018). Interparental conflict and children's social problems: insecurity and friendship affiliation as cascading mediators. Dev Psychol.

[24] Davies PT, Thompson MJ, Hentges RF, Parry LQ, Sturge-Apple ML (2022). Interparental conflict as a quadratic predictor of children's reactivity to interparental conflict and school adjustment: steeling effects or risk saturation?. Child Dev.

[25] DeLay D, Ha T, Van Ryzin M, Winter C, Dishion TJ (2016). Changing friend selection in middle school: a social network analysis of a randomized intervention study designed to prevent adolescent problem behavior. Prev Sci.

[26] Ding Y, Li D, Li X, Xiao J, Zhang H, Wang Y (2020). Profiles of adolescent traditional and cyberbullying and victimization: the role of demographic, individual, family, school, and peer factors. Comput Hum Behav.

[27] Dorio NB, Clark KN, Demaray MK, Doll EM (2019). School climate counts: a longitudinal analysis of school climate and middle school bullying behaviors. Int J Bullying Prev.

[28] Dou K, Lin XQ, Wang YJ (2020). Negative parenting and risk-taking behaviors in Chinese adolescents: Testing a sequential mediation model in a three-wave longitudinal study. Child Youth Serv Rev.

[29] Dou K, Wang LX, Cheng DL, Li YY, Zhang MC (2022). Longitudinal association between poor parental supervision and risk-taking behavior: the role of self-control and school climate. J Adolesc.

[30] Duell N, Steinberg L (2020). Differential correlates of positive and negative risk taking in adolescence. J Youth Adolesc.

[31] Duell N, Steinberg L, Icenogle G, Chein J, Chaudhary N, Di Giunta L, Dodge KA, Fanti KA, Lansford JE, Oburu P, Pastorelli C, Skinner AT, Sorbring E, Tapanya S, Uribe Tirado LM, Alampay LP, Al-Hassan SM, Takash HMS, Bacchini D, Chang L (2018). Age patterns in risk taking across the world. J Youth Adolesc.

[32] Eldik WM, Haan AD, Arends LR, Prinzie P (2020). Moderation of associations between interparental stress and (mal)adaptation by adolescents' personality: contrasting differential susceptibility and diathesis-stress models. J Pers.

[33] Farrell AD, Thompson EL, Mehari KR (2017). Dimensions of peer influences and their relationship to adolescents’ aggression, other problem behaviors and prosocial behavior. J Youth Adolesc.

[34] Figner B, Mackinlay RJ, Wilkening F, Weber EU (2009). Affective and deliberative processes in risky choice: age differences in risk taking in the Columbia Card Task. J Exp Psychol Learn Mem Cogn.

[35] Fosco GM, Bray BC (2016). Profiles of cognitive appraisals and triangulation into interparental conflict: implications for adolescent adjustment. J Fam Psychol.

[36] Fosco GM, Feinberg ME (2018). Interparental conflict and long-term adolescent substance use trajectories: the role of adolescent threat appraisals. J Fam Psychol.

[37] Fosco GM, Sloan CJ, Fang S, Feinberg ME (2022). Family vulnerability and disruption during the COVID-19 pandemic: prospective pathways to child maladjustment. J Child Psychol Psychiatry.

[38] Freeman C, Dirks M, Weinberg A (2020). Neural response to rewards predicts risk-taking in late but not early adolescent females. Dev Cogn Neurosci.

[39] Graupensperger S, Benson AJ, Bray BC, Evans MB (2019). Social cohesion and peer acceptance predict student-athletes' attitudes toward health-risk behaviors: a within- and between-group investigation. J Sci Med Sport.

[40] Grills AE, Ollendick TH (2003). Multiple informant agreement and the anxiety disorders interview schedule for parents and children. J Am Acad Child Adolesc Psychiatry.

[41] Gullone E, Moore S, Moss S, Boyd C (2000). The adolescent risk-taking questionnaire. J Adolesc Res.

[42] Harman HH (1976). Modern factor analysis.

[43] Hess S (2021). Effects of inter-parental conflict on children’s social well-being and the mediation role of parenting behavior. Appl Res Qual Life.

[44] Holbert RL, Stephenson MT (2002). Structural equation modeling in the communication sciences, 1995–2000. Hum Commun Res.

[45] Hsu YT, Kawachi I (2019). Timing of family adversity during adolescence and its impact on alcohol and tobacco initiation: a longitudinal study among Taiwanese adolescents. Child Psychiatry Hum Dev.

[46] Jia J, Li D, Li X, Zhou Y, Wang Y, Sun W, Zhao L (2018). Peer victimization and adolescent internet addiction: the mediating role of psychological security and the moderating role of teacher-student relationships. Comput Hum Behav.

[47] Jouriles EN, McDonald R, Kouros CD, Cicchetti D (2016). Interparental conflict and child adjustment. Developmental psychopathology: risk, resilience, and intervention.

[48] Ju C, Wu R, Zhang B, You X, Luo Y (2020). Parenting style, coping efficacy, and risk-taking behavior in Chinese young adults. J Pac Rim Psychol.

[49] Kerig PK (1996). Assessing the links between interparental conflict and child adjustment: the conflicts and problem-solving scales. J Fam Psychol.

[50] Koçak A, Mouratidis A, Sayıl M, Kındap-Tepe Y, Uçanok Z (2017). Interparental conflict and adolescents’ relational aggression and loneliness: the mediating role of maternal psychological control. J Child Fam Stud.

[51] Lagattuta KH, Sayfan L, Bamford C (2012). Do you know how I feel? Parents underestimate worry and overestimate optimism compared to child self-report. J Exp Child Psychol.

[52] Lei H, Zhang Q, Li X, Yang H, Du W, Shao J (2019). Cumulative risk and problem behaviors among Chinese left-behind children: a moderated mediation model. Sch Psychol Int.

[53] Li D, Li X, Wang Y, Zhao L, Bao Z, Wen F (2013). School connectedness and problematic internet use in adolescents: a moderated mediation model of deviant peer affiliation and self-control. J Abnorm Child Psychol.

[54] Li J, Li D, Jia J, Li X, Wang Y, Li Y (2018). Family functioning and internet addiction among adolescent males and females: a moderated mediation analysis. Child Youth Serv Rev.

[55] Li X, Newman J, Li D, Zhang H (2016). Temperament and adolescent problematic internet use: the mediating role of deviant peer affiliation. Comput Hum Behav.

[56] Liu Y, Li D, Jia J, Zhou Y, Zhao L, Wang Y, Sun W (2021). Perceived school climate and problematic internet use among Chinese adolescents: psychological insecurity and negative peer affiliation as mediators. Psychol Addict Behav.

[57] López-Larrosa S, Mendiri P, Sánchez-Souto V (2019). Exploring the relationship between interparental conflict and emotional security: what happens with adolescents in residential care compared to those living with their families?. Child Youth Serv Rev.

[58] Loukas A, Cance JD, Batanova M (2016). Trajectories of school connectedness across the middle school years. Youth Soc.

[59] Lux U, Walper S (2019). A systemic perspective on children's emotional insecurity in relation to father: links to parenting, interparental conflict and children's social well-being. Attach Hum Dev.

[60] Martin MJ, Conger RD, Robins RW (2019). Family stress processes and drug and alcohol use by Mexican American adolescents. Dev Psychol.

[61] Mastrotheodoros S, Van der Graaff J, Deković M, Meeus WHJ, Branje SJT (2019). Interparental conflict management strategies and parent-adolescent relationships: disentangling between-person from within-person effects across adolescence. J Marriage Fam.

[62] McCoy SS, Dimler LM, Samuels DV, Natsuaki MN (2019). Adolescent susceptibility to deviant peer pressure: does gender matter?. Adolesc Res Rev.

[63] Mehus CJ, Forster M, Chan G, Hemphill SA, Toumbourou JW, McMorris BJ (2018). Longitudinal, reciprocal relationships between family management and antisocial peer associations. J Adolesc.

[64] Merino L, Herrero M, Martínez-Pampliega A (2022). Interparental conflict appraisals and adolescents’ maladaptation in siblings: an actor-partner interdependence model. J Child Fam Stud.

[65] Muthén LK, Muthén BO (2012). Mplus user's guide.

[66] Peng S, Zhou B, Wang X, Zhang H, Hu X (2020). Does high teacher autonomy support reduce smartphone use disorder in Chinese adolescents? A moderated mediation model. Addict Behav.

[67] Pereyra SB, Bean RA (2017). Latino adolescent substance use: a mediating model of inter-parental conflict, deviant peer associations, and parenting. Child Youth Serv Rev.

[68] Petraitis J, Flay BR, Miller TQ (1995). Reviewing theories of adolescent substance use: organizing pieces in the puzzle. Psychol Bull.

[69] Podsakoff PM, MacKenzie SB, Lee JY, Podsakoff NP (2003). Common method biases in behavioral research: a critical review of the literature and recommended remedies. J Appl Psychol.

[70] Preacher KJ, Hayes AF, Hayes AF, Slater MD, Snyder LB (2008). Contemporary approaches to assessing mediation in communication research. The Sage sourcebook of advanced data analysis methods for communication research.

[71] Preacher KJ, Kelley K (2011). Effect size measures for mediation models: quantitative strategies for communicating indirect effects. Psychol Methods.

[72] Rudolph KD, Lansford JE, Agoston AM, Sugimura N, Schwartz D, Dodge KA, Pettit GS, Bates JE (2014). Peer victimization and social alienation: predicting deviant peer affiliation in middle school. Child Dev.

[73] Shin JH, Hong JS, Yoon J, Espelage DL (2014). Interparental conflict, parenting behavior, and children's friendship quality as correlates of peer aggression and peer victimization among aggressor/victim subgroups in South Korea. J Interpers Violence.

[74] Siraj R, Najam B, Ghazal S (2021). Sensation seeking, peer influence, and risk-taking behavior in adolescents. Educ Res Int.

[75] Steiger JH (1990). Structural model evaluation and modification: an interval estimation approach. Multivar Behav Res.

[76] Steinberg L, Monahan KC (2007). Age differences in resistance to peer influence. Dev Psychol.

[77] Sudraba V, Millere A, Deklava L, Millere E, Zumente Z, Circenis K, Millere I (2015). Stress coping strategies of drug and alcohol addicted patients in Latvia. Proc Soc Behav Sci.

[78] Sullivan K, Zhu Q, Wang C, Boyanton D (2021). Relations among peer victimization, aggression, and school climate among elementary school students in China. School Psychol Rev.

[79] Sznitman SR, Romer D (2014). Student drug testing and positive school climates: testing the relation between two school characteristics and drug use behavior in a longitudinal study. J Stud Alcohol Drugs.

[80] Teng Z, Bear GG, Yang C, Nie Q, Guo C (2020). Moral disengagement and bullying perpetration: a longitudinal study of the moderating effect of school climate. School Psychol.

[81] Thompson EL, Mehari KR, Farrell AD (2020). Deviant peer factors during early adolescence: cause or consequence of physical aggression?. Child Dev.

[82] Thompson MJ, Davies PT, Hentges RF, Sturge-Apple ML (2022). Delineating the developmental sequelae of children's risky involvement in interparental conflict. Dev Psychopathol.

[83] van Dijk R, van der Valk IE, Deković M, Branje S (2020). A meta-analysis on interparental conflict, parenting, and child adjustment in divorced families: examining mediation using meta-analytic structural equation models. Clin Psychol Rev.

[84] van Eldik WM, de Haan AD, Parry LQ, Davies PT, Luijk M, Arends LR, Prinzie P (2020). The interparental relationship: meta-analytic associations with children's maladjustment and responses to interparental conflict. Psychol Bull.

[85] Varela JJ, Sánchez PA, De Tezanos-Pinto P, Chuecas J, Benavente M (2021). School climate, bullying and mental health among Chilean adolescents. Child Indic Res.

[86] Walker DA, Holtfreter K (2019). Teen pregnancy, depression, and substance abuse: the conditioning effect of deviant peers. Deviant Behav.

[87] Wang LX, Dou K, Li JB, Zhang MC, Guan JY (2021). The association between interparental conflict and problematic internet use among Chinese adolescents: testing a moderated mediation model. Comput Hum Behav.

[88] Wang MT (2009). School climate support for behavioral and psychological adjustment: testing the mediating effect of social competence. Sch Psychol Q.

[89] Wang Z, Liu C, Li T, Zhao F (2020). Paternal parenting and depressive symptoms among adolescents: A moderated mediation model of deviant peer affiliation and school climate. Child Youth Serv Rev.

[90] Weymouth BB, Fosco GM, Mak HW, Mayfield K, LoBraico EJ, Feinberg ME (2019). Implications of interparental conflict for adolescents' peer relationships: a longitudinal pathway through threat appraisals and social anxiety symptoms. Dev Psychol.

[91] Xu F, Chen X, Xing H, Wang H (2021). Interparental conflict and Chinese children’s social development. J Fam Issues.

[92] Yang C, Sharkey JD, Reed LA, Dowdy E (2020). Cyberbullying victimization and student engagement among adolescents: does school climate matter?. School Psychol.

[93] Yoon D (2020). Peer-relationship patterns and their association with types of child abuse and adolescent risk behaviors among youth at-risk of maltreatment. J Adolesc.

[94] Yoon D, Snyder SM, Yoon S (2020). Child maltreatment types and adolescent substance use: the role of deviant peer affiliation. Child Fam Soc Work.

[95] Yoon D, Snyder SM, Yoon S, Coxe KA (2020). Longitudinal association between deviant peer affiliation and externalizing behavior problems by types of child maltreatment. Child Abuse Neglect.

[96] Yoon D, Yoon S, Yoon M, Snyder SM (2019). Developmental trajectories of deviant peer affiliation in adolescence: associations with types of child maltreatment and substance use. Child Youth Serv Rev.

[97] Zhai B, Li D, Li X, Liu Y, Zhang J, Sun W, Wang Y (2020). Perceived school climate and problematic internet use among adolescents: mediating roles of school belonging and depressive symptoms. Addict Behav.

