# Supplementary material for: Longitudinal association between interparental conflict and risk-taking behavior among Chinese adolescents: testing a moderated mediation model
Source: Child Adolesc Psychiatry Ment Health. 2023 Jan 10;17:5. doi: 10.1186/s13034-023-00556-4 (PMC9830742; doi:10.1186/s13034-023-00556-4)
Supplement: Supplementary file 1 — Additional file 1: Online supplementary files. Additional file 1 includes description and specific items for the four scales used in this study. Specifically, these include interparental conflict scale, deviant peer affiliation scale, school climate scale, and risk-taking behavior scale. Table 1. The means, standard deviations, correlation of school climate dimensions with the main variables. Table 2. Summary of the moderated mediation model (the model was constructed by using each of the 7 dimensions of school climate as moderating variables). [file 13034_2023_556_MOESM1_ESM.docx]

**Longitudinal association between interparental conflict and risk-taking behavior among Chinese adolescents: Testing a moderated mediation model**

**Online supplementary files**

**(1) Interparental Conflict Scale**

| **Instructions:**  This questionnaire is about the relationship between your parents. Please read each question carefully and choose one of the four options from "1" to "4" according to your experience and circle it to show how often do your parents involve in the following behaviors when they disagree. Please answer truthfully. | **Never** | **Seldom** | **Sometimes** | **Very often** |
| --- | --- | --- | --- | --- |
| 1. Call each other names. | 1 | 2 | 3 | 4 |
| 2. Threaten each other. | 1 | 2 | 3 | 4 |
| 3. Shout at each other. | 1 | 2 | 3 | 4 |
| 4. Insult (or disrespect) each other. | 1 | 2 | 3 | 4 |
| 5. Tell each other to shut up. | 1 | 2 | 3 | 4 |
| 6. Say mean words to each other. | 1 | 2 | 3 | 4 |
| 7. Break things or throw things in a quarrel. | 1 | 2 | 3 | 4 |

**(2) Deviant Peer Affiliation Scale**

| **Instructions:**  How many friends exhibit deviant behavior frequently? Deviant peer behavior includes the following behaviors, please circle the number that best fits your actual situation. | **None** | **One** | **Two to three** | **Four to five** | **More than six** |
| --- | --- | --- | --- | --- | --- |
| 1. Fights. | 1 | 2 | 3 | 4 | 5 |
| 2. Truancy or absenteeism. | 1 | 2 | 3 | 4 | 5 |
| 3. Lie. | 1 | 2 | 3 | 4 | 5 |
| 4. Internet addiction. | 1 | 2 | 3 | 4 | 5 |
| 5. Stealing. | 1 | 2 | 3 | 4 | 5 |
| 6. Smoking. | 1 | 2 | 3 | 4 | 5 |
| 7. Alcohol abuse. | 1 | 2 | 3 | 4 | 5 |
| 8. Gambling. | 1 | 2 | 3 | 4 | 5 |
| 9. Cheating in exams. | 1 | 2 | 3 | 4 | 5 |
| 10. Vandalism. | 1 | 2 | 3 | 4 | 5 |

**(3) School Climate Scale**

| **Instructions:**  Please read the following description carefully. Choose the appropriate number according to your first feeling. There is no right or wrong answer. Please do not miss any questions, even if you are not sure. | **Strongly disagree** | **Disagree** | **Agree** | **Strongly agree** |
| --- | --- | --- | --- | --- |
| 1. Most students in the class are attentive. | 1 | 2 | 3 | 4 |
| 2. School rules are fair. | 1 | 2 | 3 | 4 |
| 3. School is safe. | 1 | 2 | 3 | 4 |
| 4. Rules are made clear to students. | 1 | 2 | 3 | 4 |
| 5. Most students try their best to be the best (in study, activity, etc.). | 1 | 2 | 3 | 4 |
| 6. Teachers care about their students. | 1 | 2 | 3 | 4 |
| 7. Consequences of breaking rules are fair. | 1 | 2 | 3 | 4 |
| 8. In this school, students threaten and bully other students. | 1 | 2 | 3 | 4 |
| 9. Students know what they should do in school. | 1 | 2 | 3 | 4 |
| 10. Students are friendly towards most other students. | 1 | 2 | 3 | 4 |
| 11. In this school, bullying the weak has become a campus problem. | 1 | 2 | 3 | 4 |
| 12. Students are worried about being bullied by others at school. | 1 | 2 | 3 | 4 |
| 13. Students know what the rules are. | 1 | 2 | 3 | 4 |
| 14. Students really care about each other. | 1 | 2 | 3 | 4 |
| 15. Teachers listen to students' doubts and difficulties. | 1 | 2 | 3 | 4 |
| 16. The school's code of conduct is fair. | 1 | 2 | 3 | 4 |
| 17. Students feel safe in this school. | 1 | 2 | 3 | 4 |
| 18. The school clearly informed the students about how they should behave. | 1 | 2 | 3 | 4 |
| 19. Adults working in schools care about their students. | 1 | 2 | 3 | 4 |
| 20. Most of the students abide by the school rules. | 1 | 2 | 3 | 4 |
| 21. Students know they are safe in this school | 1 | 2 | 3 | 4 |
| 22. Most students hand in their homework. | 1 | 2 | 3 | 4 |
| 23. The classroom rules are fair. | 1 | 2 | 3 | 4 |
| 24. Most of the students study hard to get good grades. | 1 | 2 | 3 | 4 |
| 25. Students treat each other with respect. | 1 | 2 | 3 | 4 |
| 26. Students get along with each other. | 1 | 2 | 3 | 4 |
| 27. Students like their teachers. | 1 | 2 | 3 | 4 |
| 28. Teachers like their students. | 1 | 2 | 3 | 4 |

**(4) Risk-Taking Behavior Scale**

| **Instructions:**  Here are some statements about risky behavior. Please read each question carefully and choose one of the five options from "0" to "4" according to your actual situation or idea and circle it to show how much you are involved in the following behaviors. You can only choose one answer for each question. Please do not choose more than one answer or omit one answer. | **Never** | **Rarely** | **Sometimes** | **Often** | **Always** |
| --- | --- | --- | --- | --- | --- |
| 1. Leaving school. | 0 | 1 | 2 | 3 | 4 |
| 2. Underage drinking. | 0 | 1 | 2 | 3 | 4 |
| 3. Smoking. | 0 | 1 | 2 | 3 | 4 |
| 4. Getting drunk. | 0 | 1 | 2 | 3 | 4 |
| 5. Taking drugs. | 0 | 1 | 2 | 3 | 4 |
| 6. Staying out late. | 0 | 1 | 2 | 3 | 4 |
| 7. Cycling after drinking. | 0 | 1 | 2 | 3 | 4 |
| 8. Having unprotected sex. | 0 | 1 | 2 | 3 | 4 |
| 9. Overeating. | 0 | 1 | 2 | 3 | 4 |
| 10. Teasing and picking on people. | 0 | 1 | 2 | 3 | 4 |
| 11. Cheating. | 0 | 1 | 2 | 3 | 4 |

**(5) Table 1**

*The means, standard deviations, correlation of school climate dimensions with the main variables*

|  | *M* | *SD* | 1 | 2 | 3 | 4 | 5 | 6 | 7 | 8 | 9 | 10 | 11 | 12 |
| --- | --- | --- | --- | --- | --- | --- | --- | --- | --- | --- | --- | --- | --- | --- |
| **Key variables** |  |  |  |  |  |  |  |  |  |  |  |  |  |  |
| 1. Teacher-student relations | 3.10 | .51 |  |  |  |  |  |  |  |  |  |  |  |  |
| 2. Student-student relations | 3.05 | .55 | .72^***^ |  |  |  |  |  |  |  |  |  |  |  |
| 3. Student engagement-school-wide | 3.08 | .51 | .68^***^ | .64^***^ |  |  |  |  |  |  |  |  |  |  |
| 4. Clarity of expectations | 3.02 | .48 | .72^***^ | .66^***^ | .71^***^ |  |  |  |  |  |  |  |  |  |
| 5. Fairness of rules | 3.10 | .55 | .76^***^ | .68^***^ | .70^***^ | .71^***^ |  |  |  |  |  |  |  |  |
| 6. School safety | 3.11 | .54 | .75^***^ | .67^***^ | .67^***^ | .71^***^ | .88^***^ |  |  |  |  |  |  |  |
| 7. Bullying school-wide | 1.98 | .75 | -.19^***^ | -.24^***^ | -.11^*^ | -.09^*^ | -.20^***^ | -.26^***^ |  |  |  |  |  |  |
| 8. School climate (Total average score) | 3.07 | .43 | .88^***^ | .84^***^ | .83^***^ | .83^***^ | .89^***^ | .88^***^ | -.38^***^ |  |  |  |  |  |
| 9. T1 Interparental conflict | 1.63 | .60 | -.15^***^ | -.17^***^ | -.16^***^ | -.15^***^ | -.11^**^ | -.09^*^ | 0.05 | -.16^***^ |  |  |  |  |
| 10. T2 Deviant peer affiliation | 1.60 | .66 | -.28^***^ | -.23^***^ | -.20^***^ | -.27^***^ | -.29^***^ | -.27^***^ | 0.04 | -.29^***^ | .09^*^ |  |  |  |
| 11. T1 Risk-taking behavior | .39 | .34 | -.15^**^ | -.13^**^ | -.09^*^ | -.13^**^ | -.15^***^ | -.14^**^ | 0.03 | -.15^**^ | .21^***^ | .35^***^ |  |  |
| 12. T2 Risk-taking behavior | .42 | .38 | -.23^***^ | -.22** | -.12^**^ | -.21^***^ | -.18^***^ | -.21^***^ | 0.07 | -.22^***^ | .17^***^ | .40^***^ | .49^***^ |  |
| 13. T3 Risk-taking behavior | .41 | .36 | -.29^***^ | -.28** | -.18^***^ | -.23^***^ | -.22^***^ | -.25^***^ | 0.01 | -.26^***^ | .25^***^ | .39^***^ | .48^***^ | .63^***^ |

***Note*:** Sample size ranged from 513 to 550 due to missing data. ^*^ *p* < .05, ^**^ *p* < .01, ^***^*p* < .001. T1 = Time 1, T2 = Time 2, T3 = Time 3.

**(6) Table 2**

*Summary of the moderated mediation model* *(the model was constructed by using each of the 7 dimensions of school climate as moderating variables)*

|  | Model fit indices | | | | | | |  | T3 Risk-taking behavior | | | |
| --- | --- | --- | --- | --- | --- | --- | --- | --- | --- | --- | --- | --- |
|  | *χ*^2^ | *df* | *p* | RMSEA | 90% CI | CFI | SRMR |  | *B* | *SE* | β | *p* |
| **Model** |  |  |  |  |  |  |  | **Covariates** |  |  |  |  |
| Model 1 | 194.967 | 45 | <.001 | .082 | [0.070,0.093] | .864 | .072 | Teacher-student relations | -.06 | .04 | -.09 | .127 |
|  |  |  |  |  |  |  |  | Teacher-student relations×Deviant peer affiliation | -.05 | .11 | -.10 | .632 |
| Model 2 | 205.558 | 45 | <.001 | .084 | [0.073,0.096] | .853 | .071 | Student-student relations | **-.06** | **.03** | **-.11** | **.039** |
|  |  |  |  |  |  |  |  | Student-student relations×Deviant peer affiliation | -.06 | .13 | -.11 | .617 |
| Model 3 | 198.625 | 45 | <.001 | .083 | [0.071,0.094] | .861 | .072 | Student engagement-school-wide | -.03 | .04 | -.04 | .436 |
|  |  |  |  |  |  |  |  | Student engagement-school-wide×Deviant peer affiliation | -.07 | .14 | -.11 | .587 |
| Model 4 | 192.511 | 45 | <.001 | .081 | [0.069,0.093] | .866 | .071 | Clarity of expectations | -.01 | .04 | -.01 | .885 |
|  |  |  |  |  |  |  |  | Clarity of expectations×Deviant peer affiliation | -.07 | .12 | -.12 | .535 |
| Model 5 | 186.310 | 45 | <.001 | .079 | [0.068,0.091] | .872 | .070 | Fairness of rules | -.01 | .03 | -.01 | .825 |
|  |  |  |  |  |  |  |  | Fairness of rules×Deviant peer affiliation | -.06 | .10 | -.12 | .521 |
| Model 6 | 190.690 | 45 | <.001 | .080 | [0.069,0.092] | .869 | .070 | School safety | -.03 | .03 | -.05 | .347 |
|  |  |  |  |  |  |  |  | School safety×Deviant peer affiliation | -.04 | .10 | **-**.07 | .715 |
| Model 7 | 148.695 | 45 | <.001 | .068 | [0.056,0.080] | .896 | .050 | Bullying school-wide | -.04 | .02 | -.091 | .055 |
|  |  |  |  |  |  |  |  | Bullying school-wide×Deviant peer affiliation | **-.14** | **.06** | **-.213** | **.015** |

***Note****.* T3 = Time 3; The significant results are in bold. All models had interparental conflict as the independent variable, deviant peer affiliation as the mediating variable, and risk-taking behavior(T3) as the dependent variable, and controlled for baseline levels of risk-taking behavior (T1/T2). Seven models were constructed using each of the seven dimensions of school climate as moderating variables.
